# The Skaergaard trough layering: sedimentation in a convecting magma chamber

**DOI:** 10.1007/s00410-018-1466-1

**Published:** 2018-04-25

**Authors:** Z. Vukmanovic, M. B. Holness, K. Monks, J. C. Ø. Andersen

**Affiliations:** 1 Department of Earth Sciences, University of Cambridge, Downing Street, Cambridge, CB2 3EQ UK; 2 Camborne School of Mines, College of Engineering, Mathematics and Physical Sciences, University of Exeter, Penryn Campus, Penryn, Cornwall TR10 9FE UK

**Keywords:** The Skaergaard intrusion, Trough layering, Texture analysis, EBSD

## Abstract

The upper parts of the floor cumulates of the Skaergaard Intrusion, East Greenland, contain abundant features known as troughs. The troughs are gently plunging synformal structures comprising stacks of crescentic modally graded layers with a sharply defined mafic base that grades upward into plagioclase-rich material. The origin of the troughs and layering is contentious, attributed variously to deposition of mineral grains by magmatic currents descending from the nearby walls, or to in situ development by localised recrystallisation during gravitationally-driven compaction. They are characterised by outcrop-scale features such as mineral lineations parallel to the trough axis, evidence of erosion and layer truncation associated with migration of the trough axis, and disruption of layering by syn-magmatic slumping. A detailed microstructural study of the modal trough layers, using electron backscatter diffraction together with geochemical mapping, demonstrates that these rocks do not record evidence for deformation by either dislocation creep or dissolution–reprecipitation. Instead, the troughs are characterised by the alignment of euhedral plagioclase crystals with unmodified primary igneous compositional zoning. We argue that the lineations and foliations are, therefore, a consequence of grain alignment during magmatic flow. Post-accumulation amplification of the modal layering occurred as a result of differential migration of an unmixed immiscible interstitial liquid, with upwards migration of the Si-rich conjugate into the plagioclase-rich upper part of the layers, whereas the Fe-rich immiscible conjugate remained in the mafic base. Both field and microstructure evidence support the origin of the troughs as the sites of repeated deposition from crystal-rich currents descending from the nearby chamber walls.

**Electronic supplementary material:**

The online version of this article (10.1007/s00410-018-1466-1) contains supplementary material, which is available to authorized users.

## Introduction

The many possible mechanisms by which rhythmic layering forms in mafic intrusions are not well-understood, despite a wealth of publications on the subject (recently reviewed by Namur et al. 2015). Much of the difficulty in reaching a consensus on the mode of formation of any particular type of rhythmic layering derives from the fact that many studies do not involve the synthesis of detailed observations from outcrop- down to the grain-scale. In this contribution, we address the specific example of trough layering in the Skaergaard intrusion of East Greenland; a particularly contentious example of rhythmic layering that has generated a dichotomous set of interpretations in the literature since their first description by Wager and Deer (1939). We build on the detailed observational work of Wager and Deer (1939) [with further details summarised by Wager and Brown (1968)] and the many studies Neil Irvine has published on the subject (Irvine and Stoeser 1978; Irvine 1983; Irvine et al. 1998), taking advantage of the relatively recent application of electron backscatter diffraction (EBSD) to igneous rocks (e.g., Morales et al. 2011; Satsukawa et al. 2013) to generate a detailed picture of grain-scale fabrics that can then be tied to field observations. By building a comprehensive picture of the evidence preserved in the rocks themselves, we argue that the trough bands formed during sedimentation in a convecting magma chamber. This conclusion lays a solid foundation for the use of these structures in constraining the fluid dynamical behaviour of a cohesive granular medium in an igneous system.

## Geological background

**Fig. 1:**
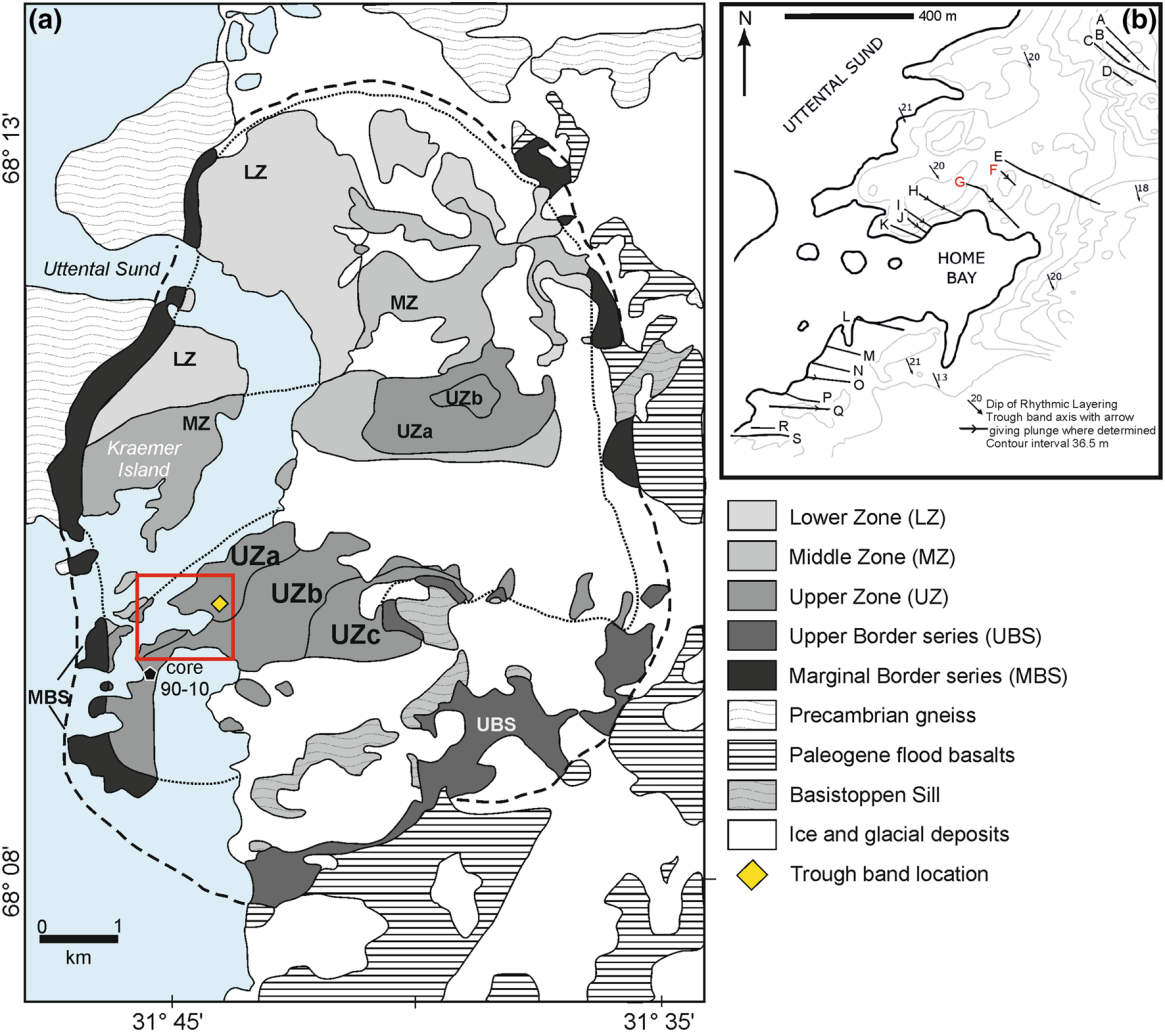
**a** Simplified geological map [after McBirney (1989)] of the Skaergaard intrusion, showing the position of the trough layering examined here, together with the location of drill core 90–10. The red box shows the area enlarged in **b. b** Map showing the location and trend of the trough axes [from Wager and Deer (1939)]

The Skaergaard intrusion (Fig. 1) formed from the injection of a body of relatively evolved tholeiitic basalt into a fault-bounded (Irvine et al. 1998) space forming on the extending margin of East Greenland during the opening of the North Atlantic. The intrusion lies at the unconformity between Precambrian gneisses and an overlying sequence of Eocene plateau lavas (Wager and Deer 1939) to which the Skaergaard magma is closely related (Nielsen 2004; Jakobsen et al. 2010). Once the chamber inflated to its final size, it remained closed both to further magma replenishment and to eruption, crystallising to form one of the world’s best examples of extreme fractionation of basaltic magma (Wager and Deer 1939; McBirney 1996).

Solidification resulted in the formation of three different series, first defined by Wager and Deer (1939): the (volumetrically dominant) Layered Series crystallised upwards from the floor; the Marginal Border Series crystallised inwards from the (vertical) walls: and the Upper Border Series crystallised downwards from the roof. The Layered Series and Upper Border Series met at the Sandwich Horizon. Progressive fractionation within the chamber resulted in these three series displaying a correlated series of changes in liquidus assemblage and permitting the subdivision of each. The Layered Series is divided into Lower, Middle and Upper Zones based on the absence of cumulus olivine in the Middle Zone. The Lower Zone is subdivided into LZa (containing cumulus olivine and plagioclase), LZb (with cumulus augite), LZc (with cumulus Fe–Ti oxides). Upper Zone is also subdivided; the base of UZb defines the arrival of cumulus apatite, while the base of UZc marks the first appearance of the mosaic form of ferro-hedenbergite inverted from β-ferrobustamite. The Upper Border Series (Salmonsen and Tegner 2013; although see; Naslund 1984) and the Marginal Border Series (Hoover 1989) can be similarly subdivided.

During the last stages of solidification, an extensive hydrothermal circulation system began to operate, particularly in the highly fractured plateau lavas that form the roof of the intrusion and the upper part of the eastern wall, leading to significant re-setting of oxygen and hydrogen isotope ratios in the uppermost parts of the intrusion in the southeast (Taylor and Forester 1979).

**Fig. 2:**
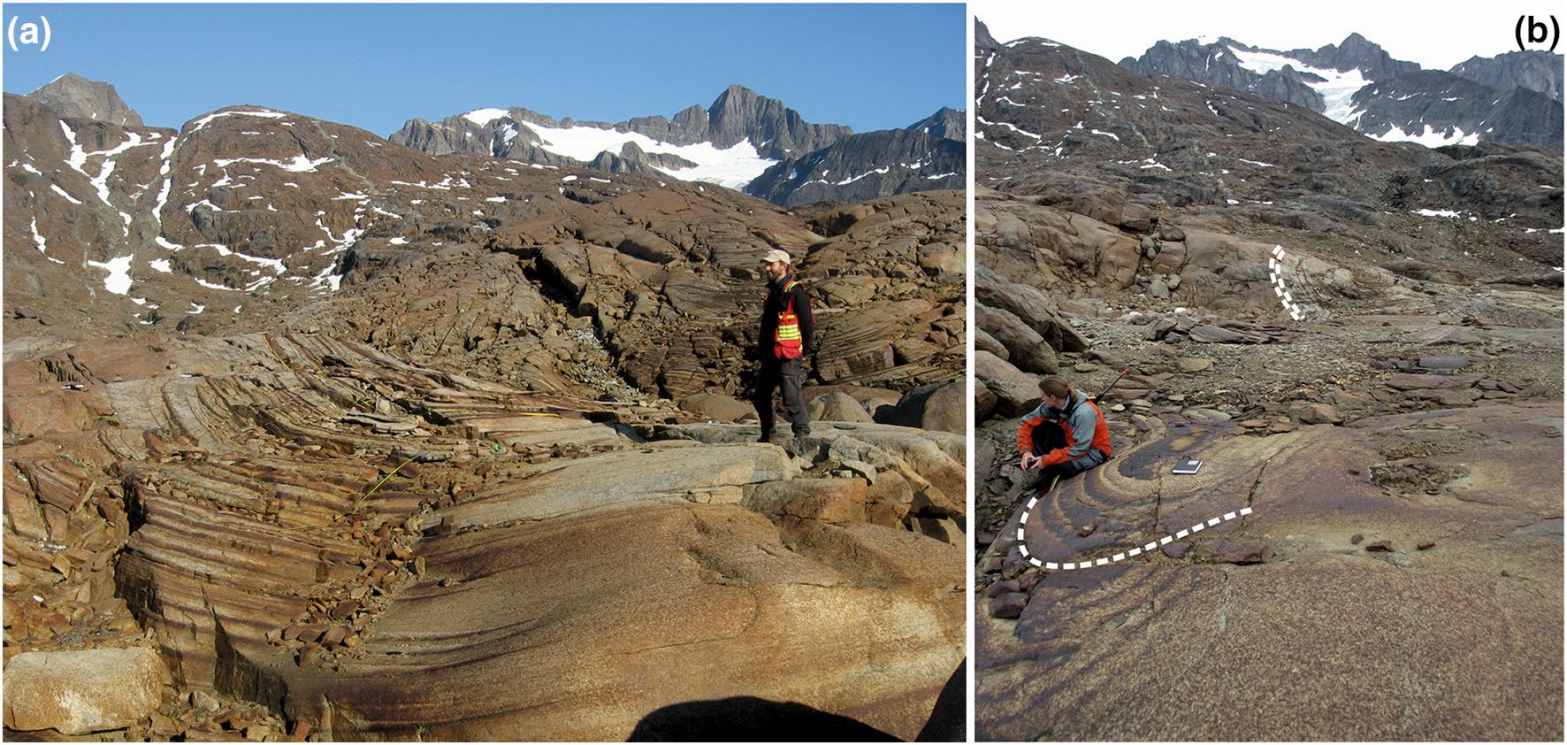
**a** Trough E, showing the stacked, crescentic, modally graded layers. Note that the layers extend for varying distances into the surrounding homogeneous unbanded gabbro. **b** View of Trough G looking along the axis towards the intrusion centre. In the foreground, the modal layers are dominated by mafic phases (oxides, olivine and clinopyroxene), whereas in the middle ground, the modal layers are dominated by plagioclase. The trough dies out towards the background

The western outcrop of the upper part of UZa is notable for the series of gently plunging synformal structures known as troughs (Wager and Deer 1939). These features are 10–50 m wide, with axial planes approximately perpendicular to the chamber walls (the trends of which are shown in Fig. 1b), and are separated by elongate mounds of massive, unlayered or poorly layered ferrogabbro (Irvine 1983). The troughs comprise stacks of upwardly concave, modally graded, layers with sharply defined mafic bases grading upwards into plagioclase-rich material (Fig. 2a). The stacks may be comprised entirely of successive modally graded layers, or contain layers of uniform gabbro that separate the modally graded layers. The contacts separating the modally graded layers from the under- and overlying uniform gabbro are usually sharply defined. The graded layers are thickest in the axis of the trough, and the mafic part of the modally graded layers generally extends further from the trough axis than the upper leucocratic component. Some troughs can be traced for ~ 300 m along strike and for more than 100 m stratigraphically (Irvine and Stoeser 1978).

Wager and Deer (1939) presented a map of the distribution of the troughs in the vicinity of their base house, in which the individual trough structures are labelled, from north to south, with a letter from A to S: they report that another group (un-labelled) is present ~ 300 m to the NE of bands A–D [shown figuratively in their Fig. 12, with photographed examples presented by Wager and Brown (1968) (their Figs. 56 and 58). This labelling scheme, developed for convenient description in the Wager and Deer (1939) memoir, is still in use, although it is far from comprehensive: Irvine and Stoeser (1978) identified a subsidiary trough south of H, and Irvine (1983) identified two further troughs (one south of D and the other south of G), together with 23 subsidiary troughs in the region between bands A and K [subsidiary troughs are those with relatively poorly defined modal layering (Irvine 1983)].

While best developed in UZa in the region shown in Fig. 1, trough structures are found throughout the intrusion, with a small number of poorly developed examples in MZ, LZc and LZa reported from the western part of the intrusion (Wager and Brown 1968) and several reported from the intrusion centre (Wager and Brown 1968; McBirney 1989).

Wager and Deer (1939) suggested that the trough layering formed by deposition of mineral grains by magmatic currents descended from the nearby walls of the intrusion and flowed across the chamber floor. The troughs were argued to mark lines of persistent strong currents and were regarded as indicating the nature of the patterns of convection in the magma chamber. This model is consistent with the observations of a preferred orientation of elongate plagioclase in the trough layers, defining a lineation parallel to the trough axes (Wager and Deer 1939; Brothers 1964; Wager and Brown 1968; Nwe 1975; Holness et al. 2017b). Following the detailed mapping work of Irvine and Stoeser (1978), Irvine (1983) developed this essentially sedimentary model by proposing that the massive ridges separating the stacks of trough layering formed during in situ crystallisation below descending parts of a series of roller-type convection cells [see Fig. 19 of Irvine (1983)]. As the ridges grew, density currents originating near the steep walls of the intrusion periodically flowed between them, depositing the modally sorted layers characteristic of the troughs.

Recently, Holness et al. (2017b) undertook a preliminary examination of three samples from the plagioclase-rich upper part of a single modal layer from Trough F, as part of a more comprehensive microstructural study aimed at constraining the extent to which viscous compaction played a role in creating the adcumulates of the Skaergaard intrusion. They conclude, in agreement with Wager and Deer (1939), that the fabrics and microstructures are consistent with a sedimentary origin.

There is, however, another school of thought, which explains the trough layers as something other than sedimentary. McBirney and Noyes (1979) stated that the trough layering lacks features typical of water- and wind-deposited sediments, with no evidence of braided structures left by shifting channels, only rare evidence of lateral migration or cutting of earlier layers, and no over-bank deposits at their margins. They, therefore, offer an alternative mechanism, suggesting that the layering is formed by localised differential diffusion during in situ growth. Taylor and Forester (1979) noted that the stratigraphic position where the troughs occur coincides with the level separating the upper parts of the intrusion which have been strongly affected by hydrothermal activity from the lower parts which retain their original stable isotopic compositions. They suggested that the ingress of water may have triggered the formation of rhythmic layering by the McBirney and Noyes mechanism. Sonnenthal (1992) argued that the trough layering was formed from initially homogeneous and unbanded gabbro by a complex, but only sketchily described, process involving the localised ingress of reactive aqueous fluids which triggered a coupled process of dissolution, compaction and flow of residual melt. This idea of in situ development of the layering by localised recrystallisation during gravitationally driven compaction was developed further by Boudreau and McBirney (1997) and McBirney and Nicolas (1997).

## Field observations and sampling

**Fig. 3:**
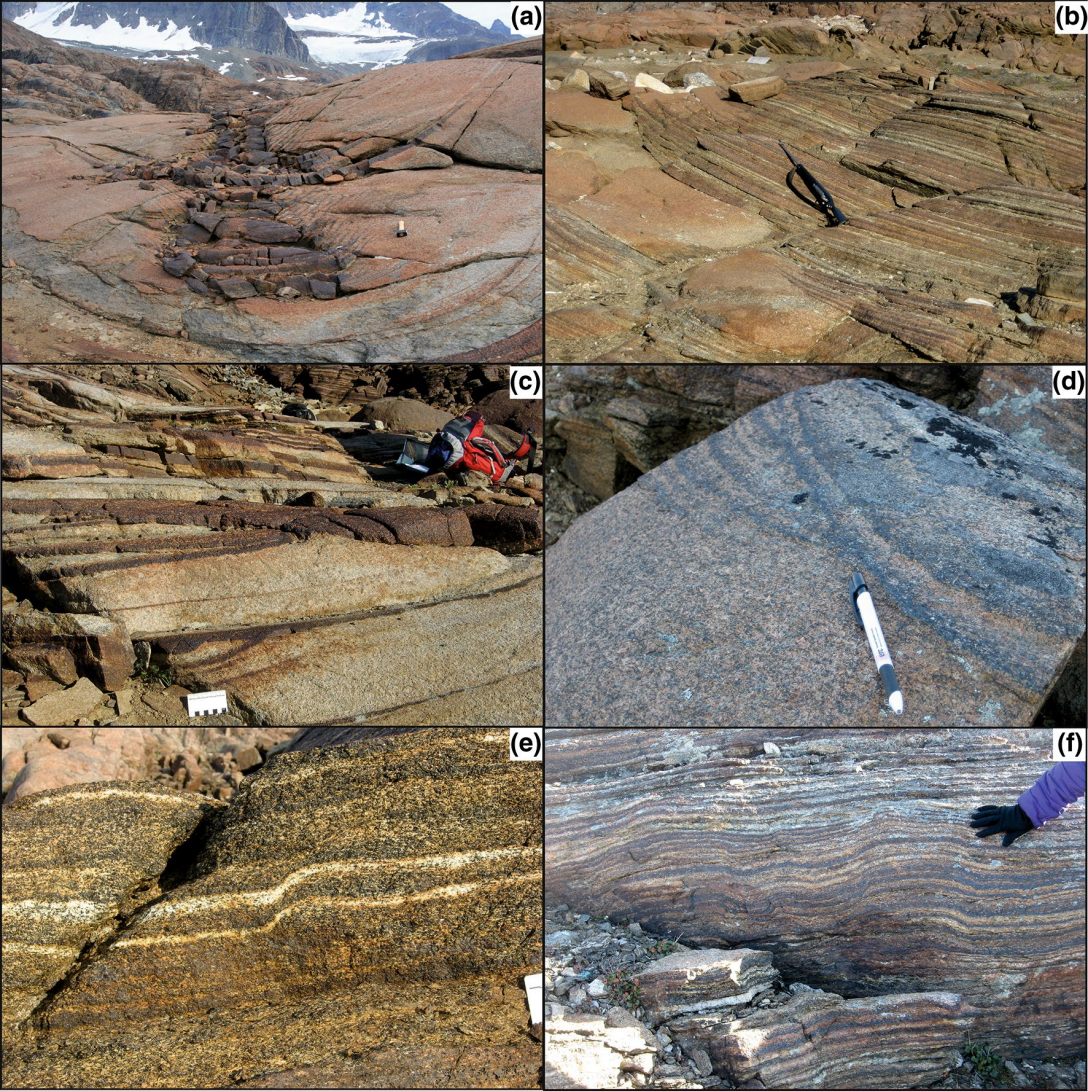
**a** Trough I, showing stratigraphic variation in the width and modal composition of the modally graded layers. Note the change from narrow, steep limbs to wider more shallow limbs, associated with erosion and truncation of the underlying narrow trough. Hammer for scale. **b** Trough O, showing temporary cessation of trough layering, followed by two stages of migration of the trough axis, with associated erosion and truncation of underlying layers. Rifle for scale. **c** A succession of events recorded in Trough E, beginning with truncation of a narrow set of modal layers by a wider set, followed by erosion and truncation of the wider set as the axis of the trough moved to the right. The scale card is marked with cm intervals. **d** A loose block from Trough O, showing clear evidence of truncation of modal layers caused by the migration of the trough axis. Pen for scale. **e** localised slumping on the southern limb of Trough S, viewed looking towards the east. The sense of slumping is consistent with migration of poorly consolidated mush towards the trough axis. Scale card on the far right shows 1 cm divisions. **f** Approximately, 60 cm of stratigraphy is formed of distorted layers in this part of Trough O. The package of distorted layers is both under- and overlain by planar, apparently undeformed layers

Irvine and Stoeser (1978) and Irvine (1983) have presented detailed descriptions of the field appearance and distribution of the UZa troughs. The stratigraphic interval containing the troughs is underlain by an interval characterised by laterally extensive planar modal layers set in homogeneous gabbro (Wager and Deer 1939; Irvine and Stoeser 1978). The troughs die out upwards, being replaced by planar, laterally extensive, modal layers. As noted by Wager and Deer (1939) [with an example mapped in detail by Irvine (1983)], individual troughs may evolve into several separate troughs with increasing stratigraphic height. Within each trough, the width and composition of the modally graded layers also changes with stratigraphic height, with intervals dominated by highly mafic layers interspersed with intervals dominated by more felsic layers (Figs. 2b, 3a). These changes in the mode of the graded layers do not correlate with any changes in the modal composition of the surrounding homogeneous gabbro, which remains constant throughout the stratigraphic interval containing the troughs.

While some modally graded trough layers form shallow crescentic shapes, others have steep limbs with the steepest parts of some limbs dipping as much as 80° [e.g., in the eastern part of Trough Band H (Wager and Deer 1939)]. The steepest (outer) parts of the limbs of Troughs F and G are locally inclined some 100° from each other. When rotated back to the palaeohorizontal, the steepest limbs of these synformal structures are inclined 40° from the horizontal.

Contrary to the comments of McBirney and Noyes (1979), there is abundant evidence of lateral migration of troughs and cutting of the underlying layers (Fig. 3b– d; Irvine 1983). Although most of the modal layers in the troughs are smoothly curved, localised examples of distorted layering are present [e.g., Figure 57 of Wager and Brown (1968)]. These distorted layers are under- and overlain by apparently undeformed layers, suggestive of syn-sedimentary slumping on the dipping surfaces (Fig. 3e, f).

**Fig. 4:**
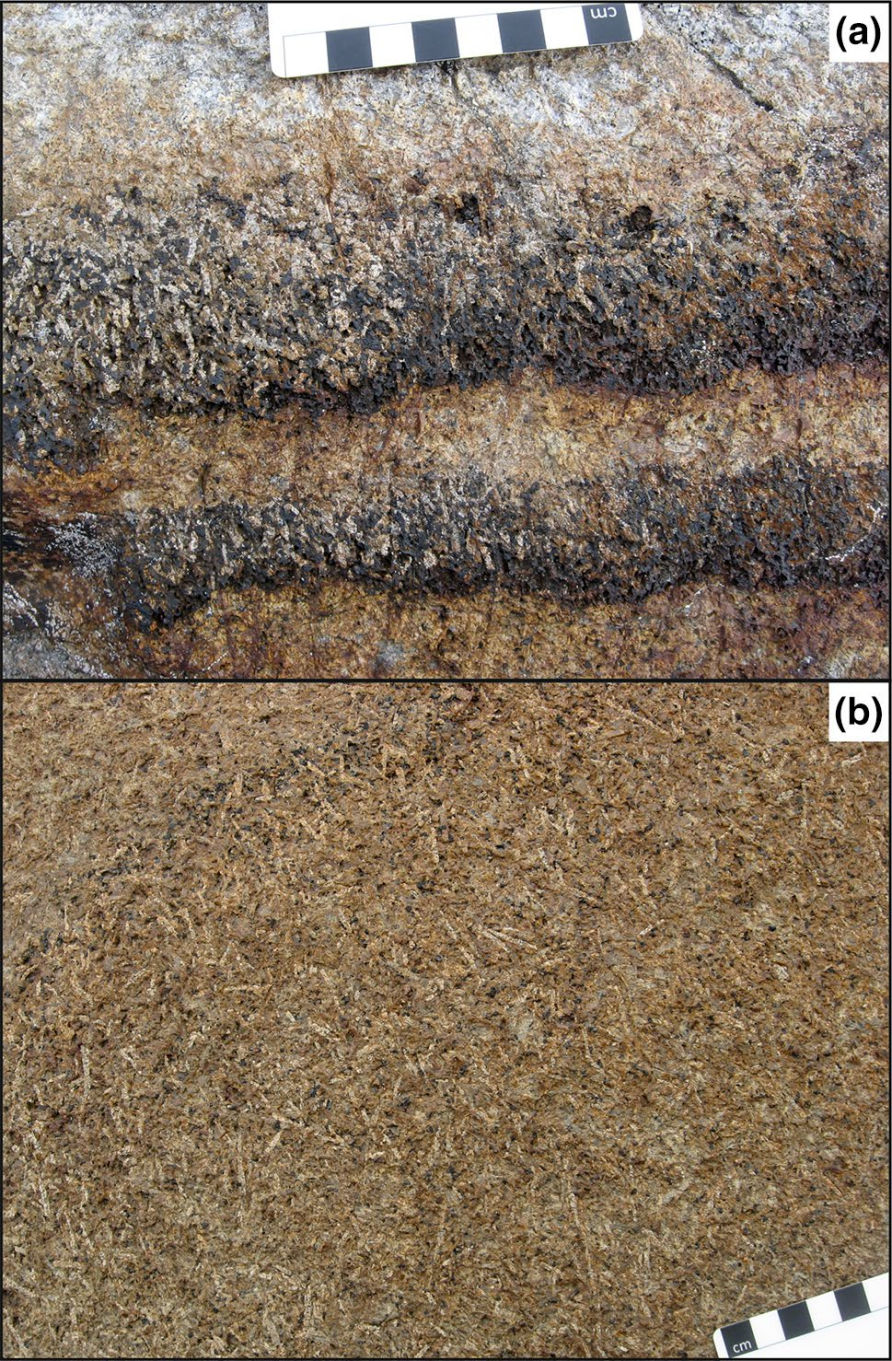
**a** the gabbro in the stratigraphic interval of UZ containing the well-developed troughs is characterised by the presence of abundant prismatic plagioclase grains that are elongated along [100]. These elongate grains form a lineation near the axes of the troughs. This example from Trough G shows the abundant elongate plagioclase lying within strongly modally graded layers that are truncated at a low angle by the outcrop surface. **b** In the planar, laterally extensive layered rocks immediately underlying the troughs, the elongate plagioclase forms a foliation. This view is looking vertically down on an exposed layering surface

The stratigraphic interval containing the troughs is notable for abundant plagioclase grains that are highly elongate (Fig. 4): these grains are scattered among the generally more equant plagioclase typical of this part of the Layered Series stratigraphy (Holness et al. 2017b). They are not present lower in the stratigraphy of the Layered Series, and become less abundant with height, disappearing together with the troughs themselves in UZb. The significance and source of the elongate plagioclase grains will be discussed in a further contribution. The elongate plagioclase grains are generally strongly aligned within the modal layers in or near the trough axes (Fig. 4a), and they form a foliation in the planar modally graded layers immediately underlying the troughs (Fig. 4b). They tend to be randomly aligned in the intervening massive gabbros.

**Fig. 5:**
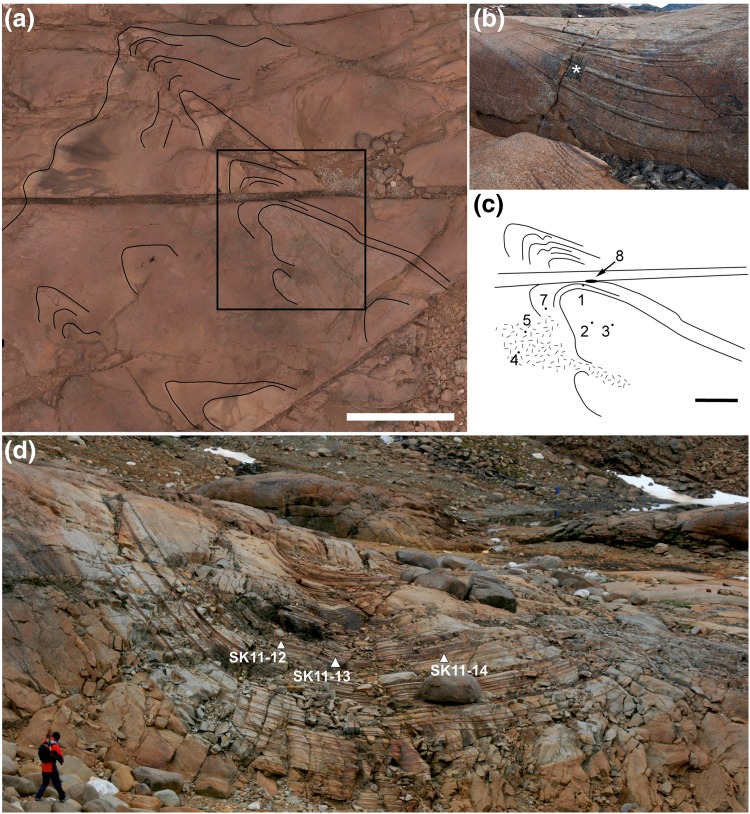
**a** Aerial view of Trough F, with North pointing upwards, taken using a drone-mounted camera. The scale bar is 20 m long. The field of view is cut by a prominent gully running E/W: this is the trace of an eroded camptonite dyke. Well-defined modal layers are outlined by the black lines, and show a poorly defined subsidiary trough to the SW of Trough F. The area outlined by the black box is shown enlarged and in schematic form in **c. b** The southern wall of the eroded camptonite dyke was sampled to collect a large specimen (F8) of the modal layering (marked with an asterisk). **c** A sketch map of the area outlined in **a**, showing schematic modal layering and the position of short drill holes collected in 1966 (F1–F7, although F6 lies out of the field of view, immediately to the west). F8 is the large specimen, with the site of collection shown in **b**. All samples are housed in the Sedgwick Museum of the University of Cambridge. **d** Trough G, viewed looking towards the intrusion centre, showing the location of the samples examined for the present study

Because the present study is aimed at determining the mechanism of formation of the modally graded trough layers, we concentrated entirely on samples from the trough themselves (both the modally graded layers and any intervening non-graded layers), with no detailed investigation of the massive gabbro found between the individual stacks of trough layers: an examination of these massive gabbros will be presented in a later study. We used a suite of samples from Trough F that was collected by G.A. Chinner during the British East Greenland Geological expedition in 1966: they are now housed in the Harker Collection of the Sedgwick Museum, University of Cambridge (Fig. 5). The suite comprises several drill cores, most of which are < 1 m long, collected from a flat-lying outcrop in the vicinity of 68°9′52″N, 31°43′12″W, and two larger specimens that were obtained from the nearby wall of an out-weathered E/W dyke by drilling a number of short cores and prising the blocks out (Fig. 5b). The first of these two blocks contains a well-developed modally graded layer, with a well-defined melanocratic base grading into a thicker felsic top (Fig. 5b). This modally graded layer is immediately overlain by a thin and diffuse (few mm thick) mafic layer which separates it from the overlying homogeneous gabbro. For the present contribution, we examined one of the 13 drill cores that was created to extract the block. We took six thin sections from the core (locations shown in Fig. 12; see later for discussion).

Four fully oriented samples were collected from Trough G, from the plagioclase-rich modal layers depicted in Fig. 5d (the same area is shown in the background in Fig. 2b). Three of these samples were analysed with electron backscatter diffraction analysis (EBSD), while the remaining sample was examined only using an optical microscope.

We also examined two samples of trough layering collected by L.R Wager, and now housed in the Harker Collection of the Sedgwick Museum, University of Cambridge (matching samples are in the collections of the Oxford Museum of Natural History). Sample 49,533 (Wager’s field number EG1493) was collected from one of the more southerly troughs on the Skaergaard Peninsula, and sample 48,969 (Wager’s field number EG2573) was collected from Trough G. No spatial information is available for these two samples.

## Methodology

### Electron backscatter diffraction analysis

Samples were prepared using the standard EBSD preparation routine described by Prior et al. (1999). Analysis was performed using a FEI sFEG XL30 SEM in the Department of Physics, University of Cambridge. Together with EBSD/SEM settings, crystallographic information for six phases (olivine, plagioclase, enstatite, diopside, ilmenite and magnetite) is presented in the Online Resource. Data collection, indexing and analysis of electron backscatter diffraction patterns (EBSDPs) were done using AZtecHKL 2.2 acquisition software. EBSD maps were constructed using Oxford Channel 5 software, whereas pole figures were plotted by MTex MatLab toolbox (Hielscher and Schaeben 2008).

The pole figure data are projected using a lower hemisphere, equal-area, projection. The fabric strength was determined by calculating the j-index and M-index of the orientation distribution function (ODF) using the MTex MatLab toolbox (Hielscher and Schaeben 2008). We calculated the j-index using the de la Vallée Pousin kernel, and a half-width of 10°, which corresponds to a series expansion of 28. The j-index has a value of one for a random distribution and a value of infinity for a single crystal (Wenk et al. 1998). The value of the M-index increases with the strength of the fabric from 0 (random fabric) to 1 (single crystal fabric) (Skemer et al. 2005). The BA index of Ulrich and Mainprice (2005) is defined by eigenvalue analysis of pole figures and describes the relative importance of (010) and [100] in the pole figures. Due to the prismatic nature of plagioclase in the trough layering, we also use the AC index that describes the relative importance of fabrics defined by alignment of (001) and [100]. The analysis of the pole figure symmetry is calculated based on P (point), G (girdle) and R (random) fabric indexes. These indexes are calculated from eigenvalues (*λ*_1_ ≥ *λ*_2_ ≥ *λ*_3_, with *λ*_1_ + *λ*_2_ + *λ*_3_ = 1) of normalized orientation matrices for the principal crystallographic axes and can be described as *P* = *λ*_1_ − *λ*_3_, *G* = 2(*λ*_2_ − *λ*_3_) and R = 3λ3 (Woodcock 1977; Vollmer 1990). The BA index is defined as 1/2[2 − {P(010)/(G(010) + P(010))} − {P[100]/(G[100] + P[100])}] (Satsukawa et al. 2013), whereas the AC is defined as 1/2[2 − {P[100]/(G[100] + P[100])} − {G(001)/(G(001) + P(001))}] (Mainprice et al. 2015). The indices range from 0 to 1.

The meaning of these indices is illustrated by their values for the three plagioclase fabric types defined by Satsukawa et al. (2013). The three Satsukawa fabric types are defined by their appearance in pole figures: an axial A fabric is defined by a strong point maximum concentration of [100] with parallel girdle distributions of (010) and (001); axial B is defined by a strong point alignment of (010) with a girdle distribution of [100]; and type P is defined by point maxima of [100], (010) and (001). The axial B fabric is characterised by a low value of BA (and AC) index (≈ 0.2), an axial A fabric by high values of BA (and AC) index (> 0.7) and the P-type fabric is defined by intermediate values. However, the interpretation of plagioclase pole figures as either a foliation or a lineation depends strongly on crystal shape (Morales et al. 2011; Ji et al. 2014; Holness et al. 2017b). All textural indices: j, M and BA were calculated with the MTex MatLab toolbox (Mainprice et al. 2015). All Euler maps were checked for systematic mis-indexing between plagioclase (010) and (001) planes: none was found.

### QEMSCAN—quantitative evaluation of minerals by scanning electron microscopy

QEMSCAN images were obtained using a Quanta 650F field emission gun equipped with two Brucker XFlash 6130 energy dispersive spectrometers (EDS) at the Department of Earth Sciences, University of Cambridge. For further information on the analysis technique and settings, see Holness (2015). The QEMSCAN images were used to create Ca concentration maps to enable easy visualisation of compositional zoning in plagioclase. Resolution of the QEMSCAN images is 5 µm.

## Petrography

**Fig. 6:**
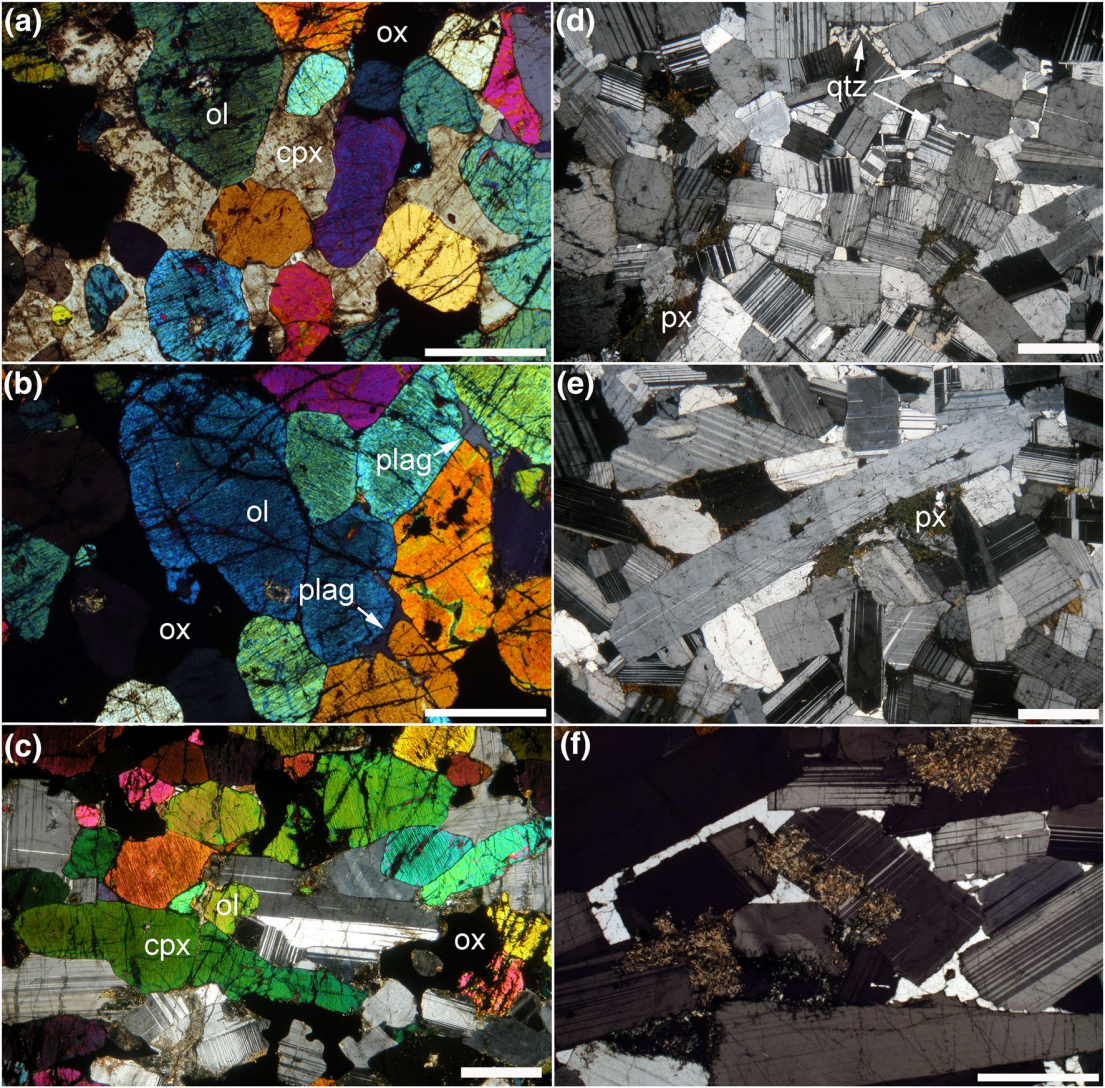
Photomicrographs of trough layering samples, all under crossed polars. **a** Sample 49,533 showing the equant and rounded shape of olivine (ol) surrounded by poikilitic clinopyroxene (cpx). Cumulus grains of Fe–Ti oxides (ox) have elongate interstitial extensions. Scale bar is 1 mm long. **b** Sample 49,533 showing granular microstructure in olivine-rich parts of the melanocratic layering. Note the small interstitial grain of plagioclase (plag—arrowed) and the interstitial patches of Fe–Ti oxide. Scale bar is 1 mm long. **c** Sample SK11-12a from Trough G, showing highly elongate clinopyroxene grains forming a foliation parallel to the modal layering. Scale bar is 1 mm long. **d** Sample SK11-12c from Trough G showing the leucocratic portion of the modal layer. Plagioclase grains are generally bounded by planar growth faces and has a low aspect ratio as viewed in thin section. Interstitial phases are quartz and an altered pyroxene (px). Scale bar is 1 mm long. **e** Sample SK11-12c from Trough G. This field of view of the leucocratic portion of the modal layer contains a highly elongate plagioclase grain which has been bent and deformed by dislocation creep. The other, more equant, plagioclase grains show no signs of deformation. Note the interstitial patches of altered pyroxene (px). Scale bar is 1 mm long. **f** Sample 48,969 showing interstitial quartz forming extensive patches in optical continuity. Note the irregular walls of the enclosing plagioclase and their compositional zoning, denoting simultaneous growth of plagioclase and quartz from an evolved Na-rich liquid in the interstitial spaces. Scale bar is 200 µm long

The basal melanocratic portion of the trough layering samples we examined is dominated by olivine, clinopyroxene and Fe–Ti oxides (Fig. 6; Table 1). The olivine is invariably rounded or subhedral (Fig. 6a), except where it forms monomineralic patches with a texturally equilibrated granular microstructure (Fig. 6b), whereas the clinopyroxene is either elongate and subhedral (Fig. 6c), or forms extensive interstitial grains enclosing the olivine (Fig. 6a). Where elongate, both olivine and clinopyroxene define a layer-parallel foliation. Fe–Ti oxides are abundant in the melanocratic layers and form large (2 mm) grains, commonly with extensive, highly irregular marginal regions that infill the interstitial spaces between the euhedral olivine (and clinopyroxene where present) (Fig. 6c). Plagioclase is rare in the melanocratic portion of the modally graded layers and is generally interstitial (Fig. 6b, c).

**Table 1:** Compositional and textural parameters of the Trough G and Trough F samples

Sample	Layer type	Mineral mode (%)				Apparent aspect ratio			Fabric parameters			
		Pl	Ol	Cpx	Ox t	Avg	Min.	Max.	J-index	M-index	BA index	AC index
Trough band G												
SK11-12A	L/M	55	17	13	15	1.85	1.77	1.94	3.9	0.06	0.72	0.66
SK11-12B	L/M	79	8	4	9	2.05	1.95	2.14	7.8	0.07	0.77	0.78
SK11-12C	L	88	0	6	6	2.03	1.95	2.12	3.9	0.09	0.71	0.60
SK11-13NA	L	78	1	17	4	2.17	2.08	2.27	2.6	0.04	0.64	0.79
SK11-13NB	M	48	13	27	12	–	–	–	–	–	–	–
SK11-13SA	L	86	0	11	3	2.12	2	2.24	–	–	–	–
SK11-13SB	L/M`	53	20	13	14	1.93	1.83	2.03	–	–	–	–
SK11-13SC	L	74	0	21	5	1.97	1.88	2.06	–	–	–	–
SK11-13SD	M	38	17	40	5	–	–	–	–	–	–	–
SK11-14A	M	58	18	13	11	–	–	–	–	–	–	–
SK11-14B	L	80	4	12	4	2.19	2.09	2.29	3.2	0.06	0.78	0.71
SK11-14C	L	81	2	11	6	2.18	2.09	2.28	3.67	0.07	0.76	0.70
Trough band F												
F8C1	HG	69	6	20	5	1.98	1.9	2.07	3.1	0.04	0.71	0.65
F8C2A	HG	69	13	13	6	2.03	1.95	2.11	4.3	0.03	0.74	0.55
F8C2B	L	92	0	4	4	2.02	1.94	2.11	3.8	0.06	0.74	0.55
F8C3	L	89	0	9	2	1.93	1.84	2.02	3.7	0.07	0.66	0.37
F8C4	M	14	39	25	22	–	–	–	–	–	–	–
F8C5	HG	67	11	16	6	1.95	1.88	2.02	4	0.06	0.75	0.55

The average apparent aspect ratio of plagioclase (Avg) is bracketed by minimum and maximum values that are the 95% confidence intervals calculated using a bootstrap method. The definitions of the fabric parameters are provided in the text

*L* leucocratic portion, *M* melanocratic portion, *HG* homogeneous gabbro. Mineral modes are volumetric (*Pl* plagioclase, *Ol* olivine, *Cpx* clinopyroxene, *Ox t* magnetite and ilmenite)

**Fig. 7:**
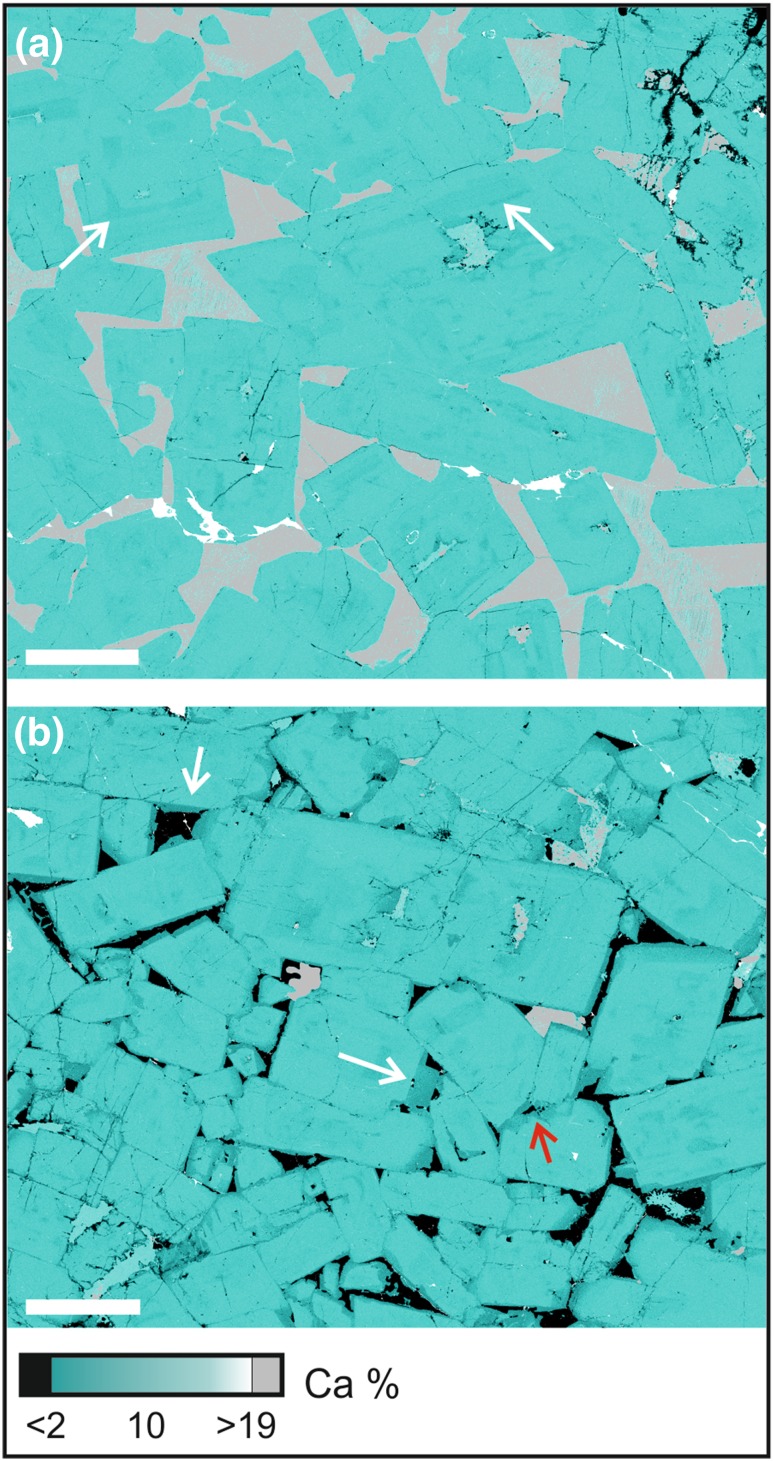
QEMSCAN maps of Ca distribution in the leucocratic portions of the modal layer sampled in Trough G. Plagioclase is teal (with darker colours showing more sodic compositions), quartz is black, and clinopyroxene is grey. The scale bar in both images is 1 mm long. **a** Sample SK11-12A. Note the concentric zoning (examples are arrowed) in the plagioclase. **b** Sample SK11-12C, showing the irregular walls of the plagioclase bounding the pockets of interstitial quartz and the sodic composition of this outermost plagioclase. The white arrows show thick relatively albitic rims that grew from evolved liquid. The red arrow shows thick compositionally zoned rims at the contacts between plagioclase crystals. Importantly, there is no preferred orientation of the late-stage overgrowths of the plagioclase grains, demonstrating that this overgrowth did not occur by dissolution–reprecipitation creep in response to an applied stress. The geometry of the plagioclase–plagioclase–quartz three-grain junctions is unmodified from the primary (disequilibrium) geometry created during solidification

The upper part of the modally graded layers is dominated by coarse-grained plagioclase (2–6 mm in length), with an average mode of 80% (Table 1). The plagioclase grains have well-developed crystal faces and the majority have a low apparent aspect ratio visible in thin section (Fig. 6d; Table 1): although the scattered grains of highly elongate plagioclase are clearly visible in hand specimen (e.g., Fig. 4), they are not sufficiently abundant to be present in large numbers in individual thin sections (Fig. 6d–f). Plagioclase grains show both oscillatory and normal zoning parallel to the crystal growth  faces (Fig. 7). The zoned rims are generally thickest where in contact with interstitial quartz (Fig. 7b), but are also present at contacts between plagioclase grains (Fig. 7b). There is no systematic pattern in the orientation of late-stage overgrowths on plagioclase. The junctions of two plagioclase grains are always formed by the impingement of planar growth faces (Fig. 7), with no evidence of either super- or sub-solidus modification of this primary igneous geometry by textural equilibration. The plagioclase is generally undeformed. The rare evidence of undulatory extinction and deformation twins is found in those grains exhibiting a high apparent aspect ratio in the plane of the thin section (Fig. 6e). We found no evidence of either subgrain formation or grain boundary migration indicative of dynamic recrystallisation.

Clinopyroxene and Fe–Ti oxides are locally present in the upper part of the modally graded layers as rare euhedral grains. Both phases are more commonly present as interstitial grains, together with small amounts of green amphibole and other late-stage hydrous minerals (Fig. 6e). Quartz is an abundant interstitial phase in the leucocratic parts of the modally graded layers (Fig. 6d, f), but is entirely absent from the melanocratic portion. Where it fills large interstitial pockets, the surrounding plagioclase is strongly normally zoned, has the irregular margins indicative of cotectic growth of plagioclase and quartz (Figs. 6f, 7b), and disequilibrium, primary three-grain junction geometries (Fig. 7b).

The layers of homogeneous gabbro that separate the modally graded layers within Trough F comprise > 65 to 70% modal plagioclase, ~ 20% modal clinopyroxene, ~ 10% olivine and ~ 5% Fe–Ti oxide (Table 1). In thin section, the plagioclase has the same shape and size as that in the leucocratic parts of the modally graded layers, and is similarly undeformed. Olivine crystals are generally smaller (~ 100 to 200 µm in diameter) compared to those in the melanocratic portion of the modally graded layers. Clinopyroxene can be both subhedral and interstitial in the same sample. Fe–Ti oxides form compact grains, commonly with a euhedral habit. Quartz fills interstitial pockets bounded by planar plagioclase faces.

**Fig. 8:**
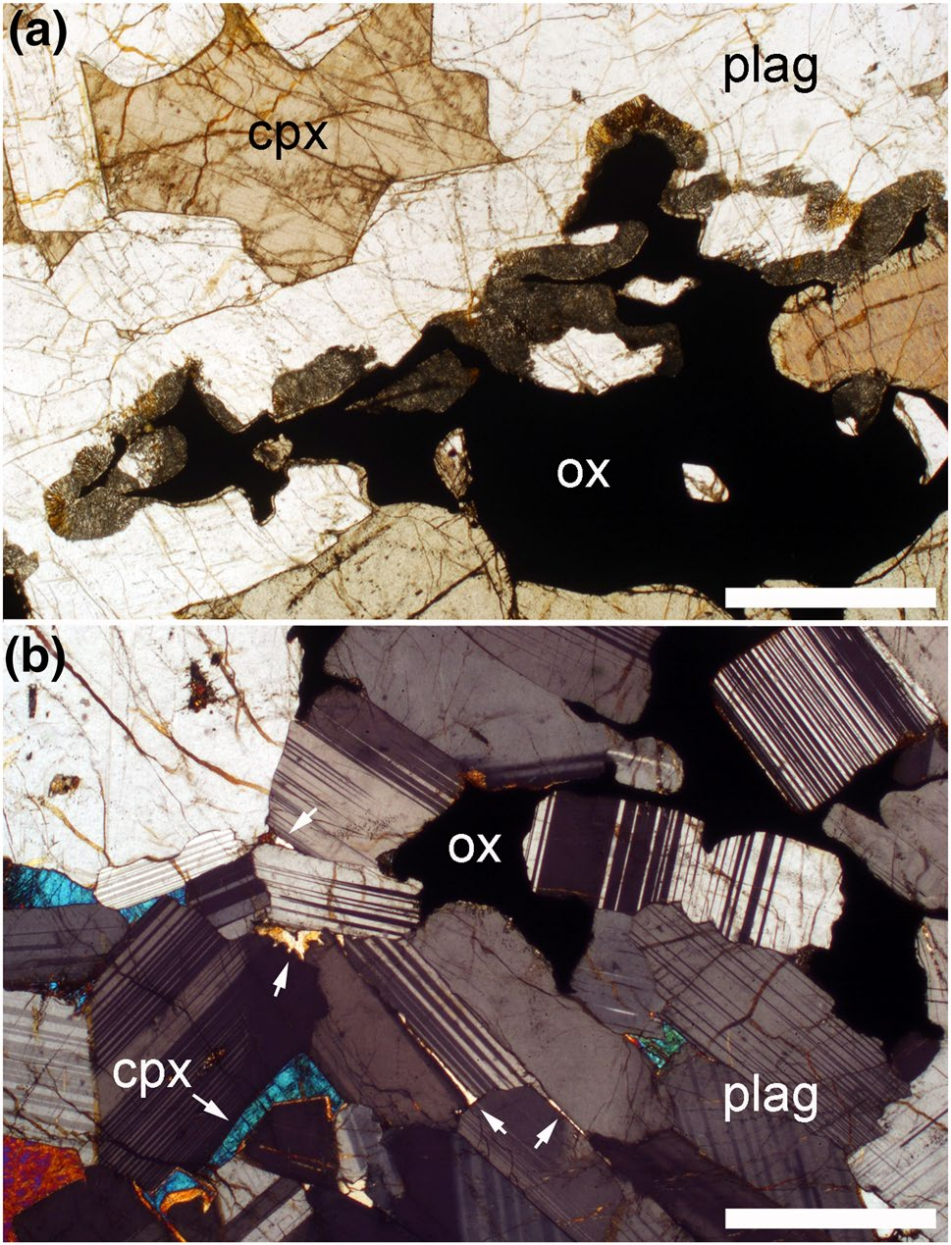
Photomicrographs of sample 48,969. The scale bar in both images is 1 mm long. **a** Extensive reactive symplectites, formed of anorthitic plagioclase and clinopyroxene (cumulus grains of clinopyroxene are labelled cpx), growing from Fe–Ti oxide grains (ox) and replacing plagioclase (plag). Symplectites are common at the margins of the melanocratic layers. **b** Interstitial Fe–Ti oxides in the leucocratic portions of the modally graded layers are not associated with reactive symplectites, but occur in association with interstitial quartz (arrowed)

As originally noted by Wager and Deer (1939, their Fig. 27), large and well-developed reactive symplectites, comprised of anorthitic plagioclase intergrown with either olivine or clinopyroxene, are common at the margins of the mafic portion of the modally graded layers (Fig. 8a). They grow outwards from Fe–Ti oxide grains and replace plagioclase. Reactive symplectites are absent from the leucocratic portions of the modally graded layers (Fig. 8b), and are uncommon in the homogeneous gabbro separating the modally graded layers in Trough F: they are entirely absent below the modally graded layer, and only locally developed near the base of the homogeneous gabbro overlying the modally graded layer (i.e., in the vicinity of the thin diffuse mafic layer).

## Plagioclase grain shape

Plagioclase grains in UZ gabbros take one of two forms. The volumetrically dominant population is almost prismatic and slightly elongated along [100] (Gay and Muir 1962; Nwe 1975; Holness et al. 2017b) (Fig. 6a), whereas a second (much smaller) population forms highly elongated prismatic grains (Fig. 6e). These unusual shapes mean that any preferred orientation forms fabrics that are not directly comparable to those defined by Satsukawa et al. (2013) who considered only the more usual tabular plagioclase flattened parallel to (010). A magmatic foliation formed by prismatic plagioclase grains is characterised by a girdle of [100] and identical point maxima of poles to (010) and (001) [the latter is caused by the equivalence of the (010) and (001) planes parallel to the foliation (Morales et al. 2011)]. The reader is referred to Holness et al. (2017b) for a summary of pole figures for shape preferred orientation (SPO) fabrics formed by prismatic plagioclase crystals.

## Fabric analysis

### Trough G

**Fig. 9:**
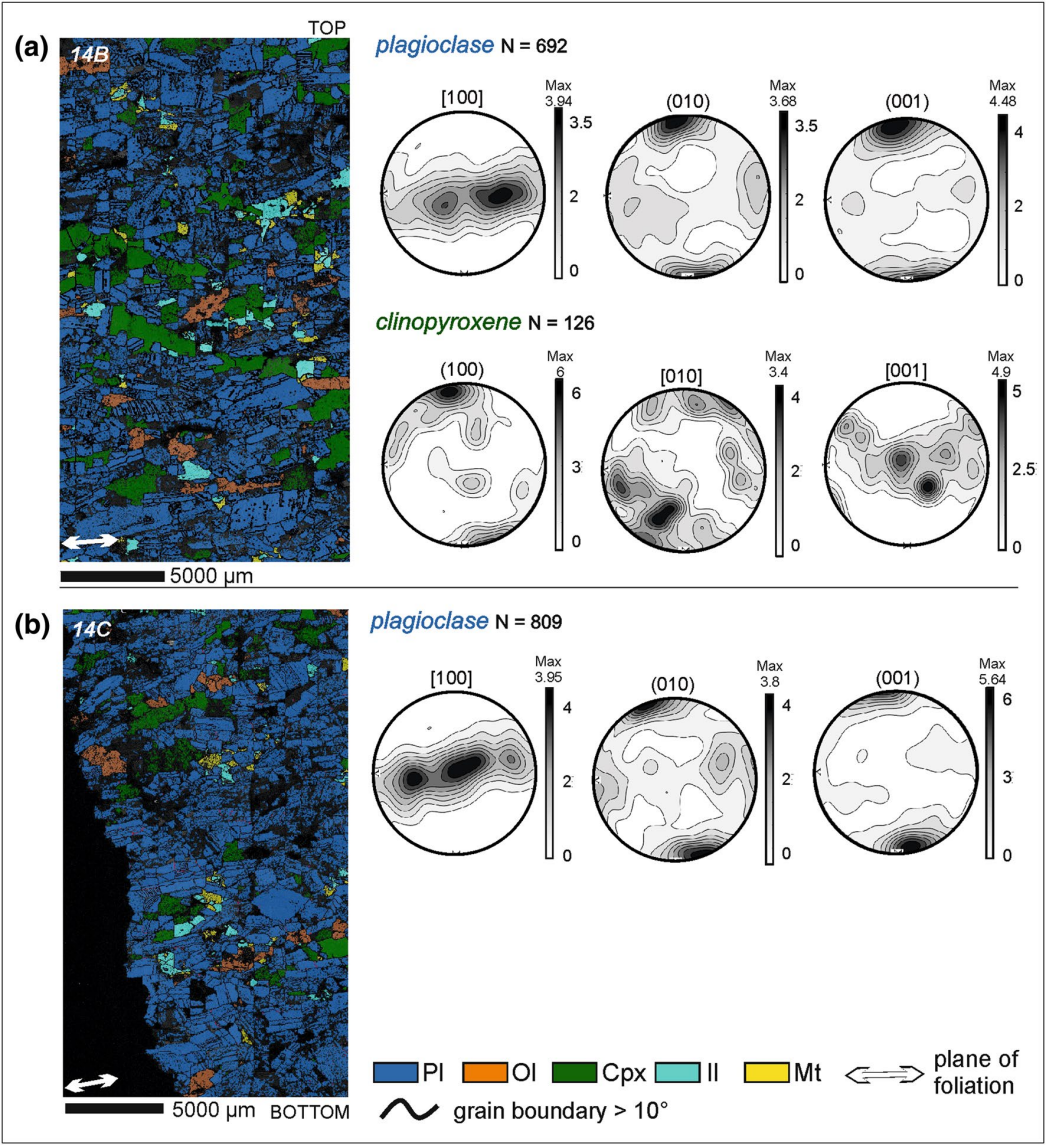
**a** Orientation and phase map of sample SK11-14B (Trough G) together with lower hemisphere equal-area projections of plagioclase and clinopyroxene orientation distribution functions (ODF) calculated from one point per grain subsets. **b** Orientation and phase map of sample SK11-14C (Trough G), together with lower hemisphere, equal-area, projections of plagioclase ODF calculated from one point per grain subsets. The orientation of the foliation is shown in each phase map by a double-headed white arrow

The leucocratic part of sample SK11-14 (from near the trough axis) shows a strong plagioclase point maximum for (001) and a weak point maximum for (010) planes (Fig. 9). The [100] axes are distributed along a girdle parallel to the foliation visible in thin section (Fig. 9). The (100) planes of interstitial clinopyroxene are also distributed along the foliation. Clinopyroxene [010] and [001] axes are distributed as a point maximum and a weak girdle, respectively (Fig. 9a). The plagioclase fabric in this leucocratic material does not correspond to any of the simplified fabrics described by Satsukawa et al. (2013), because of the unusual prismatic grain shape. The BA and AC indices are 0.6–0.7 (Table 1) and this, together with the girdle distribution of [100], is consistent with the fabric being a foliation.

**Fig. 10:**
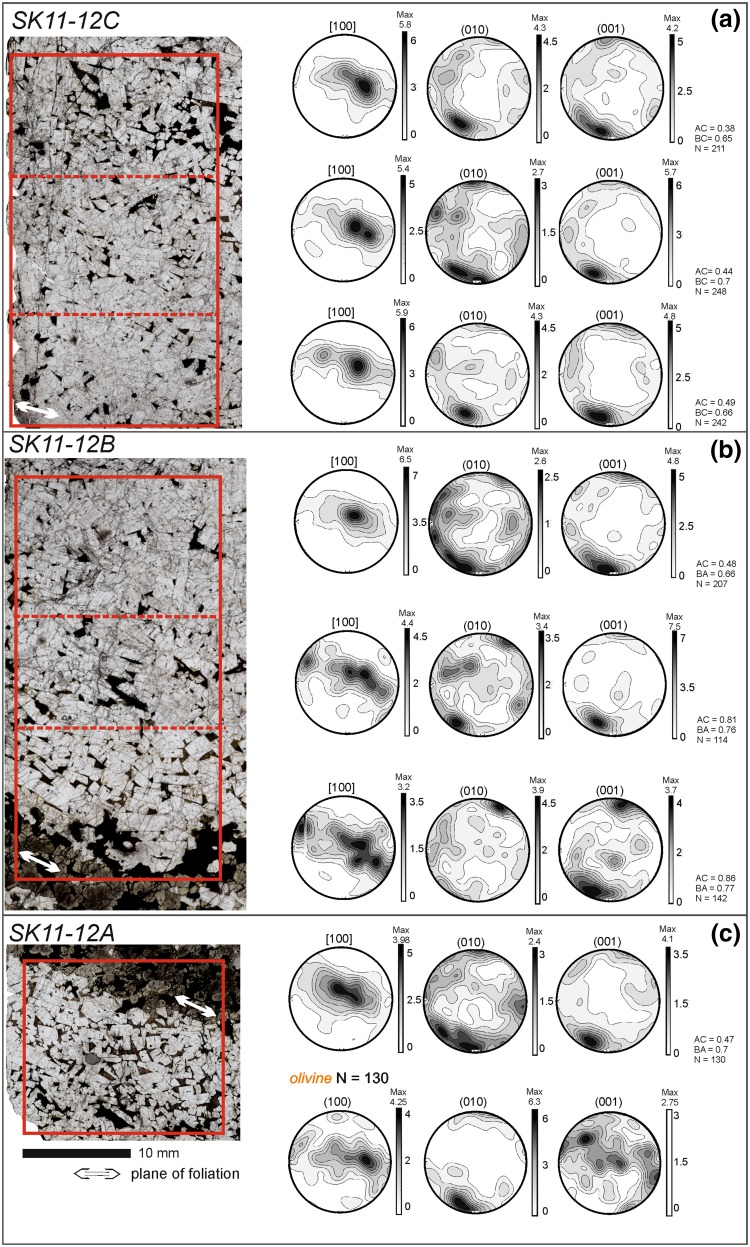
Photomicrographs of samples **a** SK11-12C, **b** SK11-12B, and **c** SK11-12A (Trough G), which form a continuous stratigraphy comprising three contiguous thin sections (oriented so that the younging direction is towards the top of the page). The orientation of the foliation is shown by the white double-headed arrow in each photomicrograph. Note the well-defined mafic band towards the base of the sampled stratigraphy—this marks the base of a single modally graded layer. The leucocratic portion of each thin section was subdivided to examine spatial variations in fabric strength and type (the different areas are shown by the red rectangles). The fabric in each of these areas is shown by lower hemisphere, equal-area, projections of plagioclase orientation distribution functions (ODF) calculated from one point per grain subsets

Sample SK11-12 (from the limb of the trough), includes a melanocratic band with the associated overlying leucocratic material, and extends downwards to include the upper part of the leucocratic material from the underlying modally graded layer (Fig. 10). The upper leucocratic material has a similar texture to SK11-14: (001) and (010) form strong point maxima with (001) being dominant. The [100] axes form a stronger elongate maximum that spreads out in the plane of the foliation (Fig. 10a). The leucocratic material just above the melanocratic portion also shows the strong point maximum of (001) and (010), but with (010) dominance (Fig. 10b). The distribution of [100] forms a weak girdle that spreads out from a strong maximum.

Although both SK11-13 (only one thin section analysed) and SK11-14 preserve a foliation-dominated fabric throughout the leucocratic portions of the modally graded layer, in sample SK11-12, the fabric changes with distance from the melanocratic layer (Figs. 9, 10; Table 1). The leucocratic material from the underlying modally graded layer preserves a lineation-dominated fabric (Fig. 10c), but there is a shift from a (010) to a (100) dominant fabric towards the top of the leucocratic material in the upper modally graded layer (Fig. 10; Table 1). The AC index is the closest to 0.5 at the top of the leucocratic layer (Fig. 10a, SK11-12C). This variation in the pole figures is consistent with a change from a foliation immediately above the melanocratic layer to a lineation at the top of the leucocratic layer.

Olivine crystals from the melanocratic layer form a fabric characterised by a girdle of [100], a strong point maximum of (010) and a weak (001) girdle (Fig. 10c). This suggests that olivine forms a foliation defined by a preferred alignment of (010) planes, parallel to the modal layering.

**Fig. 11:**
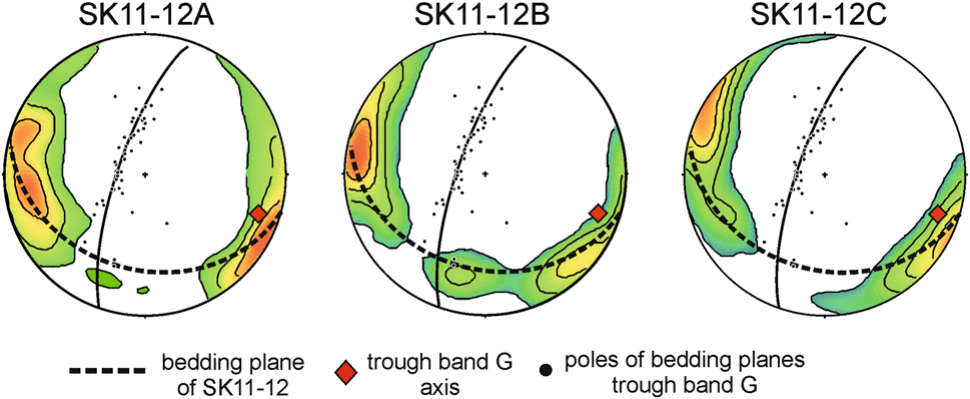
A lower hemisphere stereographic projection of poles to the modal layering in Trough G, with the EBSD orientation data of [100] for the three SK11-12 samples superimposed. The EBSD data was oriented to match the true field orientation of SK11-12

For comparison, the plagioclase pole figure distribution for the [100] axes in sample SK11-12 is plotted together with the poles to the layering observed in the field (Fig. 11). Although the number of field data points is small, the trough axis (red diamond, Fig. 11) plots close to the point maximum of the [100] axes, confirming Wager and Deer’s (1939) assertion that the plagioclase lineation is parallel to the axis of the troughs.

### Trough F

**Fig. 12:**
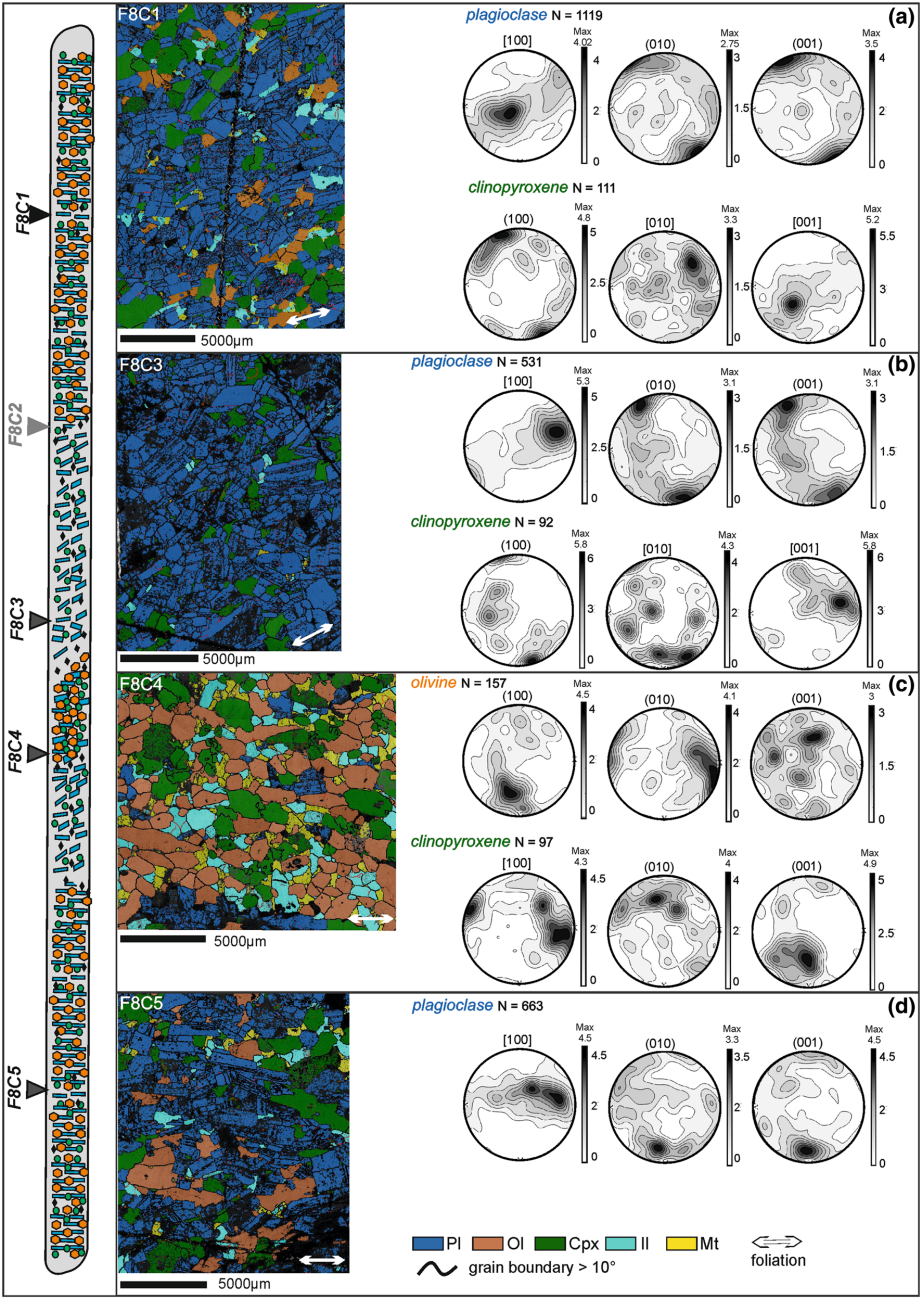
Trough F orientation data. At the extreme left of the figure is a sketch of the F8 core with arrows indicating the location of thin sections. Stratigraphic up is towards the top of the page. For each sample, the orientation and phase map is shown (with the orientation of the foliation shown by the white double-headed arrow in each phase map), together with lower hemisphere, equal-area, projections of plagioclase and clinopyroxene orientation distribution functions (ODF), each calculated from one point per grain subsets. **a** Sample F8C1—homogeneous gabbro. **b** Sample F8C2—leucocratic portion of the modally graded layer. **c** Sample F8C4—melanocratic portion of the modally graded layer. **d** Sample F8C5—homogeneous gabbro

The locations of the five analysed samples are shown in Fig. 12. Plagioclase crystals from the homogeneous gabbro overlying the modally graded layer (sample F8C1) are oriented with their [100] axes forming a point maximum parallel to the layering. The poles of (010) and (001) form point maxima perpendicular to modal layering, consistent with a weak lineation formed by prismatic plagioclase grains. Clinopyroxene forms a weaker fabric, with (100) planes parallel to the foliation, while [010] and [001] axes are distributed along weak point maxima (Fig. 12a). Plagioclase in the underlying homogeneous gabbro (sample F8C5) also forms a weak lineation, with both the [100] axis and the two poles to (010) and (001) forming point maxima (Fig. 12d). Insufficient numbers of clinopyroxene and olivine grains were analysed to obtain meaningful results.

In the leucocratic material near the base of the modally graded layer (sample F8C3), plagioclase forms a lineation, with [100] axes forming a strong point maximum perpendicular to the elongate point maxima formed by (010) and (001) (Fig. 12b). The plagioclase fabric in the leucocratic material at the top of the modally graded layer (sample F8C2, pole figures are not shown in Fig. 12) has AC values close to 0.5, also suggesting a lineation (Table 1).

In thin section and hand specimen, the fabric in the melanocratic part of the modally graded layer is defined by subhedral, slightly elongated, olivine. Olivine pole figures (sample F8C4) show that (100) and (010) form strong point maxima, whereas (001) is distributed along multiple weak point maxima (Fig. 12c). In the case of clinopyroxene, the poles to the (100) planes form a point maximum, as do both [010] and [001] axes. Hence, the melanocratic material is characterised by weak lineation of both olivine and clinopyroxene grains.

## Discussion

### The troughs as sedimentary features in a convecting magma chamber

Field evidence commonly cited in support of compaction in sedimentary rocks includes the reduction of bedding thickness of the more deformable layers (i.e., shale vs. sandstone). The combination of homogeneous mounds of massive ferrogabbro and the intervening trough layering does have some resemblance to the geometry of sedimentary beds that have been subjected to preferential compaction where they lap onto more rigid mound-like features (e.g., Carminati et al. 2010). However, crystals within the compacted beds cannot form lineations unless they are subjected to additional simple shear. Furthermore, the combination of clearly developed field-scale features such as thinning of the trough layering on the limbs of the troughs (Fig. 4a), shifting of the trough axis (Fig. 4b), layer truncations (Fig. 4c, d) and slumping confined to narrow stratigraphic intervals (Fig. 4e, f) can only be explained by sedimentary deposition involving strong directional current activity.

Only rarely do plagioclase crystals in our samples contain deformation twins: the majority of plagioclase grains are not deformed. There is no evidence for either low angle boundaries or undulatory extinction, discounting deformation by dislocation creep. The well-developed magmatic zoning, predominantly euhedral shape and absence of any preferred orientation of late-stage overgrowths on plagioclase means that deformation cannot have occurred by dissolution–reprecipitation creep either. The plagioclase forms an SPO characterised by the preferred alignment of the grain long axis [100] parallel to the modal layering, forming either a foliation or a lineation which, in the case of Trough G, is almost exactly parallel to the trough axis (Figs. 9, 10, 11). Another feature that cannot be explained by the preferential compaction model of Boudreau and McBirney (1997) and McBirney and Nicolas (1997) is the presence of abundant interstitial quartz in the leucocratic material of the modally graded layers. If these layers the result of compaction, such interstitial late-stage minerals would be more abundant in the adjacent homogeneous gabbros instead.

The following microstructures point to only minimal overprinting of the primary magmatic fabrics: inter-phase grain boundaries are dominated by those defined by low index growth faces of one or both minerals; the grain shape of the dominant minerals in the modal layers (e.g., olivine and pyroxene in the melanocratic portions and plagioclase in the leucocratic portions) is defined by growth faces; and three-grain junctions have a disequilibrium geometry. Because the first stages of recrystallisation of primary igneous microstructures involve modification of primary disequilibrium dihedral angles (Holness et al. 2005; Higgins 2011), the entirely unmodified three-grain junction geometries in the gabbros described here mean that they cannot have undergone any post-accumulation microstructural modification. This is supported by the planar shape of the grain boundaries, which would have become curved during textural equilibration. The corollary of this is that gabbros in the troughs retain their original magmatic microstructures and cannot have undergone the recrystallisation suggested by McBirney and Noyes (1979), Sonnenthal (1992), Boudreau and McBirney (1997) and McBirney and Nicolas (1997).

We, therefore, conclude that the original Wager and Deer (1939) model for the formation of the troughs is correct: they represent the site of repeated deposition of modally graded layers from crystal-rich currents descending from the nearby chamber walls and progressing across the chamber floor.

### The behaviour of immiscible interstitial liquids in the crystal mush

Germane to any model of the formation of modally graded layers by deposition by magmatic currents is an understanding of the strength of hydrodynamic sorting of the particles. Our detailed petrographic examination of the trough layers shows that the extent of modal layering present in the fully solidified rock has been modified during solidification by the post-accumulation differential migration of immiscible interstitial liquid.

Evidence for differential migration of immiscible interstitial liquids is provided by reactive symplectites such as those shown in Fig. 8 (Holness et al. 2011). These symplectites are formed when the buoyant Si-rich conjugate leaves the mushy layer, leading to a chemical destabilisation of the remaining dense Fe-rich conjugate which consequently reacts with the surrounding primocrysts. Reactive symplectites are common in the Layered Series from LZc upwards, but disappear higher in the stratigraphy, to be replaced by an assemblage of interstitial material comprising granophyres and ilmenite-rich intergrowths. These are interpreted to be the solidified remains of pockets of Si-rich and Fe-rich conjugate liquids, and their presence denotes the retention of the buoyant Si-rich conjugate in the mush. The transition between cumulates which lost their Si-rich conjugate and those that retained it is shown in Fig. 1 of McBirney (1998): it occurs at higher levels in the centre of the intrusion compared to the margins (Holness et al. 2011).

Importantly, the transition from rocks which lost their Si-rich conjugate (and therefore contain reactive symplectites) to rocks which retained their Si-rich conjugate (and therefore contain the paired assemblage of granophyre and ilmenite-rich intergrowths) occurs in MZ in drill core 90–10 [position shown in Fig. 1. NB, the figure showing the stratigraphic distribution of these late-stage features in drill core 90–10 in Holness et al. (2017a) is incorrect, although the accompanying text is correct]. This is consistent with the absence of reactive symplectites and the presence of interstitial quartz in the homogeneous gabbro underlying the modally graded layers in Trough F. In contrast, the modally graded layers in Trough F contain abundant and well-developed reactive symplectites: they are found at the margins of the melanocratic layers (Fig. 8a), but are absent from the relatively quartz-rich leucocratic layers (Fig. 8b). This suggests that there has been significant differential migration of immiscible interstitial conjugate liquids within the modally graded layer itself.

The very different behaviour in the massive gabbro and the modally graded layers can be explained as a consequence of the different wetting properties of the two immiscible conjugates. In the upper parts of the Layered Series, granophyre is almost invariably found in interstitial pockets bounded by plagioclase, whereas the ilmenite-rich intergrowths are found in pockets bounded by olivine, oxide and clinopyroxene (Holness et al. 2011). This suggests that the Si-rich liquid preferentially wets plagioclase, whereas the Fe-rich liquid preferentially wets the mafic phases and oxides. In a mush containing an immiscible emulsion, the two components of the emulsion will move to occupy pores bounded by phases on which they have a low wetting angle: in a homogeneous mush, migration of the two conjugates will be on the grain-scale and will not result in their separation on a larger scale. However, within a modally graded layer, the absence of plagioclase-bounded pores in the melanocratic portion means that the Si-rich conjugate will rise upwards, driven by capillary forces, and occupy pores in the leucocratic portion, while the Fe-rich conjugate will sink into the melanocratic portion. This cm-scale separation of the two conjugates destabilises the Fe-rich conjugate in the melanocratic portion, leading to the development of reactive symplectites, whereas the abundance of the Si-rich conjugate in the leucocratic layer means that any remaining Fe-rich liquid remains in chemical equilibrium. The presence of reactive symplectites in the homogeneous gabbro immediately overlying the leucocratic layer suggests that excess Fe-rich liquid may have ponded here, unable to move downwards due to the unfavourable wetting conditions.

These observations demonstrate that modal grading in a crystal mush, with Fe-rich minerals at the base and Si-rich minerals at the top, is likely to be amplified by differential migration of an immiscible interstitial liquid, driven by capillary forces. A true idea of the extent of hydrodynamic sorting in such a layer can be gained only from a consideration of the modal proportions of the primocrysts, rather than from bulk geochemical analyses.

## Conclusions

The conclusion that the troughs and trough layering represent a record of erosion and deposition of crystals provides immense research opportunities. It not only opens the way towards the development of understanding the behaviour of a cohesive granular medium with these particular fluid dynamical properties, but can also be used as a natural laboratory to study the behaviour of crystal mushes and hence to understand the deep crustal plumbing system under volcanoes (Cashman et al. 2017). Although we are still in the earliest stages of this work, two interesting conclusions can be drawn from the present study.

First, the troughs can potentially provide us with previously unavailable information about the likely angle of repose of a mushy layer in a cooling magmatic system. The recent work by Holness et al. (2017b), together with the results of the present contribution, suggest that compaction was insignificant for these rocks, so the present-day angle of repose is likely to be close to, or the same as, the original angle. While there appears to be no effect of liquid viscosity on the angle of repose (Carrigy 1970; Samadani and Kudrolli 2001), it will be affected by particle shape (Pohlman et al. 2006) and, perhaps most importantly, by cohesion (Samadani and Kudrolli 2001). Inter-particle cohesion in a magmatic system is likely to be considerable, as crystals sinter together to form grain boundaries (e.g., Philpotts and Carroll 1996; Philpotts et al. 1999; Philpotts and Dickson 2000). However, the modally graded trough layers and fabrics described here could only have formed if individual grains behaved essentially as discrete, non-cohesive, particles. The angle of repose of a gabbroic crystal mush might, therefore, be similar to that of poorly cohesive granular particles reported in the literature, consistent with the observation that the maximum apparent dip of the trough walls was generally ~ 40°.

Second, the Trough G leucocratic samples that are located on the limb (SK11-12) and nearer to the axis (SK11-13 and SK11-14) show different fabrics: the limb sample is lineated, whereas the axial samples are foliated (Figs. 9, 10; Table 1). While the number of samples is limited, these results hint that the slope of the terrain may influence the type of fabric created by magmatic flow. Further work should be done to elucidate this.

## Comment

We are aware of the considerable consternation in the geological community concerning the manner in which material was collected from Trough F (Bridgewater et al. 1978). In recognition of this, we made every effort during our 2017 field season to locate the source of all samples collected in 1966, and to create the detailed and accurate map that is a fundamental prerequisite for ensuring their scientific usefulness. Their accession to the Harker Collection of the Sedgwick Museum of the University of Cambridge further ensures that they remain available for examination and analysis by other scientists.

## Electronic supplementary material

Below is the link to the electronic supplementary material.

Supplementary material 1 (PDF 96 KB)
