# Supplementary material for: The Skaergaard trough layering: sedimentation in a convecting magma chamber
Source: Contrib Mineral Petrol. 2018 Apr 25;173(5):43. doi: 10.1007/s00410-018-1466-1 (PMC6560677; doi:10.1007/s00410-018-1466-1)
Supplement: Supplementary file 1 — Supplementary material 1 (PDF 96 KB) [file 410_2018_1466_MOESM1_ESM.pdf]

EBSD and SEM settings

**EBSD settings**		**SEM Settings**	
Background (frames)	64	system	Philips XL30 SFEG SEM
Binning	4 x 4		
Gain	10		
Hough resolution	60	Acc Voltage (kV)	20
Band detection (min/max)	6/7	working sitance	20
Noise reduction - wild spike'	yes	Spot size	5
n Neighbour zero solution extrapolation	5	Aperature	3
Kuwahara Filter	3 x 3 / 5°/1°	Tilt	70°

Mean Angular distribution

**Phases**	SK11-12A	SK11-12B	SK11-12C	SK11-13NA	SK11-14B	SK11-14C	F8C1	F8C2A	F8C2B	F8C3	F8C5
Plagioclase	0.78	0.76	0.77	0.78	0.87	0.78	0.79	0.82	0.8	0.74	0.8
Olivine	0.68	0.72	0.84	0.7	0.84	0.7	0.6	0.71	1.01	1.04	0.68
Clinopyroxene	0.78	1.16	0.77	0.76	0.84	0.76	0.7	0.75	0.76	0.74	0.77
Orthopyroxen	n/a	0.98	1.7	1.3	1.3	n/a	0.84	1.02	1.5	0.88	0.85
Ilmenite	0.74	0.7	0.69	0.77	0.8	0.71	0.7	0.71	0.73	0.7	0.7
Magnetite	0.7	0.78	0.68	0.8	0.83	0.72	0.75	0.76	0.89	0.75	0.76
Zero Solution	61.8	49.2	34.9	30.65	35.8	51.2	18.89	59.9	50.57	43.8	40.15
Step size (µm	20	20.25	20	20	20	20	20	20	20.25	20	20

References for the matching units

Plagioclase	American Mineralogist, (1990) vol 75. pp 150-162
Olivine	Forsterite.cry (Aztec Database)
Clinopyroxene	Diopside.cry (Aztec Database)
Orthopyroxene	Enstatite.cry (Aztec Database)
Ilmenite	Phys. Chem. Miner. (1995) col 22, 251-258
Magnetite	J.Appl. Crystallogr. 1998) vol. 31 718-725
